# Liquid biopsy for non-invasive monitoring of patients with kidney transplants

**DOI:** 10.3389/frtra.2023.1148725

**Published:** 2023-06-08

**Authors:** Anthony Nassar, Katharine Cashman, Shreya Rao, Maribel Dagher, Connor O’Brien, John Afif, Paolo Cravedi, Jamil R. Azzi

**Affiliations:** ^1^ Transplantation Research Center, Renal Division, Brigham and Women’s Hospital, Boston, MA, United States; ^2^ Division of Nephrology, Department of Medicine, Translational Transplant Research Center, Immunology Institute, Icahn School of Medicine at Mount Sinai, New York, NY, United States

**Keywords:** biomarkers, liquid biopsy, ddcfDNA, exosomes, proteomics, cytokines, kidney diseases, kidney transplantation

## Abstract

The current tools for diagnosing and monitoring native kidney diseases as well as allograft rejection in transplant patients are suboptimal. Creatinine and proteinuria are non-specific and poorly sensitive markers of injury. Tissue biopsies are invasive and carry potential complications. In this article, we overview the different techniques of liquid biopsy and discuss their potential to improve patients’ kidney health. Several diagnostic, predictive, and prognostic biomarkers have been identified with the ability to detect and monitor the activity of native kidney diseases as well as early and chronic allograft rejection, such as donor-derived cell-free DNA, exosomes, messenger RNA/microsomal RNA, proteomics, and so on. While the results are encouraging, additional research is still needed as no biomarker appears to be perfect for a routine application in clinical practice. Despite promising advancements in biomarkers, the most important issue is the lack of standardized pre-analytical criteria. Large validation studies and uniformed standard operating procedures are required to move the findings from bench to bedside. Establishing consortia such as the Liquid Biopsy Consortium for Kidney Diseases can help expedite the research process, allow large studies to establish standardized procedures, and improve the management and outcomes of kidney diseases and of kidney transplant recipients.

## 1. Introduction

Using liquid biopsy techniques for biomarker discovery research has become an emerging field for expanding diagnostic and prognostic tools for patients with kidney diseases. Generally, a liquid biopsy refers to the molecular analysis of non-tissue samples from body analytes. Body fluids, such as blood and urine, are most commonly used in these techniques, but saliva, stool, and other body fluids can also be used as sources for liquid biopsies (1).

Current clinical tools utilized for the diagnosis and management of kidney disease often fall short of demonstrating the whole picture of a patient's kidney health. In the United States alone, one in every seven people experience chronic kidney disease (CKD), and two in every five people with CKD unknowingly have severe CKD. In addition, as of March 2022, a little over 90,000 patients were on a waitlist for a kidney transplant, with only 22,817 kidney transplants taking place in the United States in 2020 (2). Of the patients fortunate enough to receive a kidney transplant, one in five will lose their allograft within 5 years and more than 50% will lose their allograft within 10 years (3). These statistics clearly emphasize the need for better tools for both the detection of native kidney disease and allograft status monitoring.

Serum creatinine and proteinuria are the two main currently utilized tools for the detection of kidney disease, but both are non-specific and often detect kidney injury too late. For transplant patients, in particular, creatinine can often remain unchanged while subclinical rejection can occur within the allograft (4). In lieu of these suboptimal clinical markers, a traditional tissue biopsy is considered the gold standard for determining disease etiology and progression. However, traditional tissue biopsies are invasive procedures that pose health risks to patients, including bleeding, infection, and, in rare cases, organ loss (5). Furthermore, there are still significant disadvantages to a tissue biopsy as a diagnostic tool, including sampling bias and intra- and inter-observer discrepancies in histopathologic analysis (6).

Given the significant patient population affected by the current suboptimal clinical tools available, there is a need for the development of more specific, less invasive approaches. Liquid biopsy techniques are one solution to this current challenge. Cancer research was among the first fields to truly start utilizing liquid biopsies for diagnosis and prognosis (7). Circulating tumor cells (CTCs) and circulating tumor DNA (ctDNA) are two of the most widely used biomarkers for cancer translational research. Their applications consist of cancer diagnostics, prognostics, and therapeutics monitoring (8). The vast benefits these liquid biopsy techniques have had on cancer research have paved the way for further research in other scientific areas. Herein, we will review data from human studies on the use of liquid biopsy techniques in kidney research.

## 2. History and techniques

The idea of a liquid biopsy has existed for over 150 years, but there has been recent interest in developing the procedure to emphasize performing preventative medical practices rather than reactive medical practices (9). Recently, there has been more research carried out for liquid biopsies used for cancer. Additional tumor products besides ctDNA and CTCs, such as circulating messenger RNA (mRNA), microRNA (miRNA), exosomes, and tumor educated platelets (TEP), can be used as biomarkers. Studies in gastrointestinal (GI) oncology show that, in fact, liquid biopsies can be used more effectively than tumor tissue samples, as tissue samples may be limited, making genomic profiling difficult. In addition, liquid biopsies can give the physicians a complete picture of the tumor burden at one specific moment in time (10). If several of these “snapshots” can be captured over a period of time, one can better understand the patient's disease, as the aforementioned biomarkers will be at heightened levels for patients with malignant cancer.

Liquid biopsies have a certain degree of sensitivity and specificity to its test, as these tests attempt to detect the alterations in the fluid instead of the volume dilution of the tumor itself (11). However, this can be improved by coupling biomarkers. For instance, prostate cancer studies can track ctDNA with prostate-specific antigen (PSA) (12).

## 3. Liquid biopsy and biomarkers in kidney transplants

**Figure 1:**
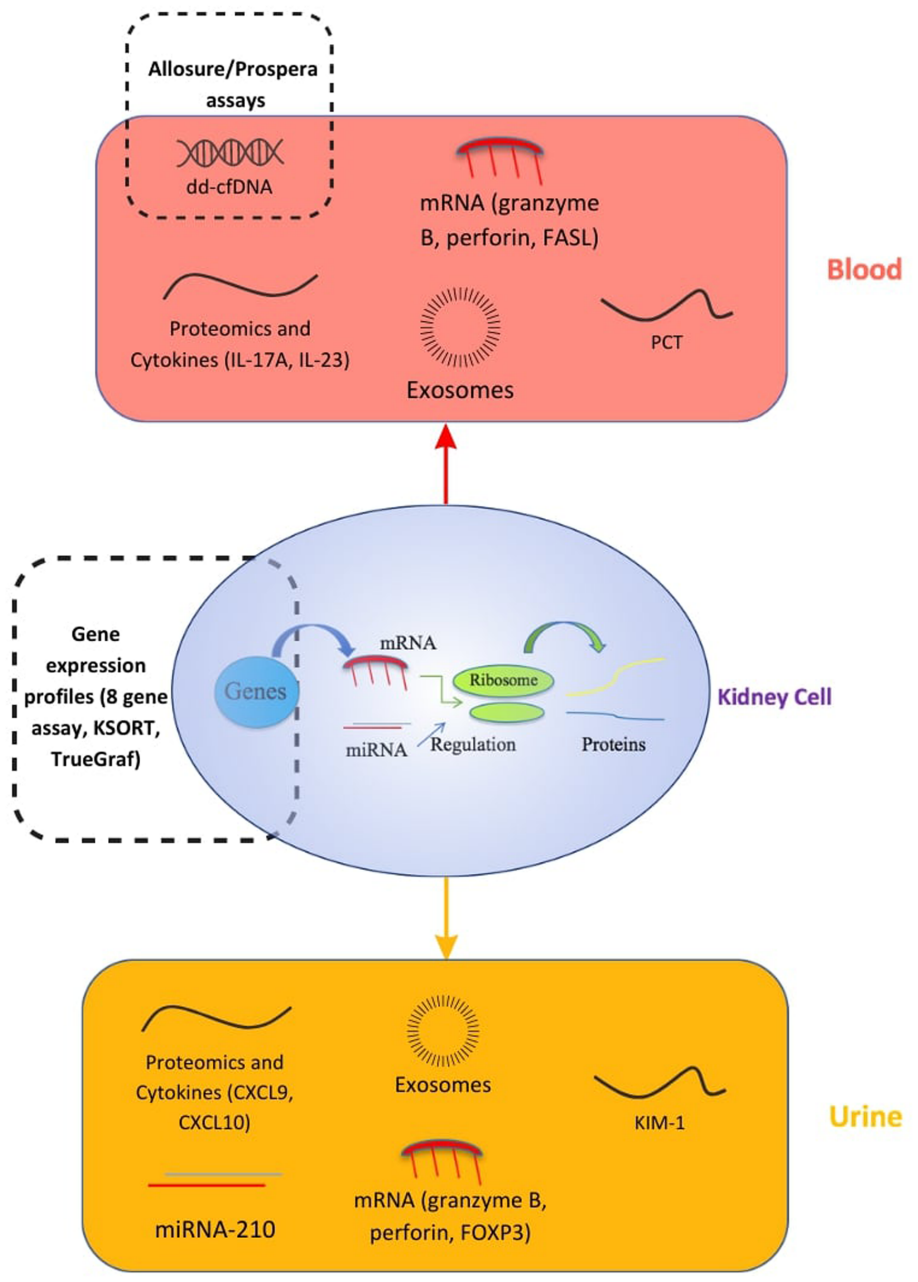
Biomarkers in kidney transplants. CXCL, C-X-C motif Ligand; ddcfDNA, donor-derived cell-free DNA; FASL, FAS Ligand; FOXP3, Forkhead box protein P3; IL, Interleukin; KIM-1, Kidney Injury Molecule 1; KSORT, Kidney Solid Organ Response Test; mRNA/miRNA, messenger RNA/microsomal RNA; PCT, procalcitonin (20).

For transplant patients, the glomerular filtration rate and proteinuria as signs of allograft rejection are non-specific, because of other immunological or non-immunological factors that can affect the graft's function (13). Kidney biopsy remains the gold standard for the early detection of graft loss. As a result, a lot of effort was deployed to research and develop biomarkers and liquid biopsy techniques as non-invasive and accurate alternatives to detect early-stage rejection and prevent allograft loss. These biomarkers, illustrated in Figure 1, can be isolated from either blood or urine. There are three types of biomarkers: diagnostic; prognostic; and predictive. A diagnostic biomarker identifies the presence of a disease or condition. A prognostic biomarker can be used to predict a clinical event, such as disease progression or recurrence, irrespective of treatments. A predictive biomarker on the other hand can change in response to treatment, and would be very useful in treatment follow-ups as it can identify patients who are most responsive to therapies (14).

### 3.1. Donor-derived cell-free DNA

Cell-free DNA (cfDNA) consists of portions of nucleic acids released in the blood and other fluids when cells go through apoptosis or necrosis. After renal transplantation, plasma donor-derived cell-free DNA (ddcfDNA) levels increase in the first few days and later decrease to become relatively stable after 10 days (15). At a steady state, the ratio of donor to recipient cell-free DNA has been shown to be less than 1%. Indeed, in the Circulating Donor-Derived Cell-free DNA in Blood for Diagnosing Acute Rejection in Kidney Transplant Recipients (DART) study, a cutoff of 1% has been established to differentiate any cause of rejection from no rejection with an area under the curve (AUC) of 0.74 (95% CI 0.61–0.86), a sensitivity of 59%, a specificity of 85%, a positive predictive value (PPV) of 61%, and a negative predictive value (NPV) of 84% (16). It has also been proven that ddcfDNA levels decline significantly after treatment for allograft rejection whereas serum creatinine levels persist unchanged. Thus, monitoring of ddcfDNA may be a useful diagnostic biomarker for assessing allograft rejection (17).

This knowledge helped put in place a number of validated tests, such as Allosure, which detects ddcfDNA using a polymerase chain reaction (PCR)-based technology with next-generation sequencing (NGS) read-out. However, Allosure helps detect antibody-mediated rejection (AMR), but is not helpful in detecting T-cell mediated rejection.

Contrary to Allosure, Prospera is another assay using the same technology, but is capable of identifying 13,000 single-nucleotide polymorphisms (vs. 266 for Allosure). This assay can be used to diagnose T-cell mediated rejection (4).

However, there are several limitations in using ddcfDNA. First, it is important to note that it is a marker of injury and not rejection. ddcfDNA is not specific for rejections and has a low PPV. Elevated levels can be caused by non-rejection allograft injuries, such as BK nephropathy, glomerulonephritis, active urinary tract infection, and even biopsy-related kidney injury if a biopsy was performed within 12 h. On the other hand, graft damage is not always associated with elevated ddcfDNA, such as in interstitial fibrosis and tubular atrophy (IFTA). Clinical context is therefore very important when interpreting a positive result (18).

Furthermore, studies showed that ddcfDNA was not able to distinguish T-cell mediated rejection IA (TCMR IA) from no rejection, which may result in false negatives at an early stage.

Finally, some patients have a slower than normal decline of ddcfDNA after transplantation, which may result in higher levels and an incorrect diagnosis of rejection (4).

### 3.2. Exosomes

Extracellular vesicles (EVs) have an important role in kidney biomarkers. There are three types of EV: exosomes; microvesicles (MVs); and apoptotic bodies. Exosomes were first discovered in 1983 in the supernatants of sheep reticulocytes. It was later found that they are generated via endosome systems and secreted by numerous cells, including mesenchymal stem cells, macrophages, and cancer cells. Exosomes are released widely in biological fluids (plasma, urine, bone marrow, and amniotic fluid), are easily accessible with a nanoscale diameter in the range of 50–150 nm, and consist of proteins (heat-shock proteins, tetraspanins, alix), lipids (ceramide, cholesterol), and nucleic acids (DNA, mRNAs, microRNAs) (19).

For example, exosomal mRNA is a useful tool in the diagnosis of a transplant rejection. It is very stable at ambient temperatures, which allows long handling, shipping, and storage time. Two urinary exosomal mRNA signatures have been identified in renal transplant rejection: one signature identified all cause rejection (AUC 0.93; NPV 93.3%; PPV 86.2%), and the second was able to distinguish between TCMR and AMR (AUC 0.87; NPV 90.6%; PPV 77.8%), as it is a molecular signature and not an injury signature like ddcfDNA (20, 21).

Plasma exosomal miRNA on another hand can also be used to monitor allograft renal function: a panel of three miRNAs (miR-21, miR-210, and miR-4639) was able to discriminate between normal renal function and chronic allograft dysfunction (CAD) (AUC 0.89, sensitivity 88.46%, and specificity 73.08%) (22).

Studies on exosomes are also limited, with a need for larger validation studies in post-transplant monitoring. While exosome extraction assays remain relatively expensive and limited to few clinical laboratories, there is a clear effort by many groups to make this technology widely available to patients, which is the case for the commercially available exosome-based prostate test (ExoDx™ prostate test) (23).

### 3.3. MicroRNAs and messenger RNA

MicroRNAs and messenger RNA can be measured in the blood or urine. miRNAs are short nucleotide sequences assuring their role of degradation of target mRNA by binding to complementary mRNA and thus promoting gene regulation. Urinary levels of miRNA-210, for example, were associated with severe allograft rejection (sensitivity 52%, specificity 74%, AUC 0.70), as well as a reduction in glomerular filtration rate (GFR) 1 year after the transplant. miRNA-210 also came back to normal after rejection treatment, and may be a very promising predictive biomarker in kidney transplant recipients. Research on miRNA in kidney diseases is limited, and there are no standard protocols for isolation and processing, which can restrict their application in clinical practice (14). mRNA is a source of biomarkers found in both blood and urine. On the one hand, in blood, mRNA levels of molecules, such as granzyme B, perforin, and Fas ligand (FASL), have been associated with acute allograft rejection and were found elevated in both the peripheral blood and graft tissue of patients with acute rejection (AR) compared to patients with no rejection (sensitivity 100%, specificity 95%, PPV 100%, NPV 95%). Likewise, urinary-cell granzyme B and perforin mRNA profiles helped differentiate between allografts with acute rejection and allografts with no acute rejection (sensitivity 79%–83%, specificity 77%–83%). In addition, Forkhead box protein P3 (FOXP3) mRNA increased expression in blood and urine is associated with biopsy-confirmed acute rejection (sensitivity 94%, specificity 95%, PPV 94%, NPV 95%, AUC 0.95). However, it is important to note that mRNA is not very stable in body fluids. These high-performance values must be interpreted carefully, given the small sample size in the studies coupled with a high prevalence of confounders in transplant patients, such as polypathology for example. The results need to be confirmed in larger validation studies (14).

### 3.4. Proteomics and cytokines

Urinary biomarkers include proteomics, which is the measurement of proteins and peptides in the urine, with 70% of them being generated from the kidney. Proteomics analysis provides opportunities to discover new urinary biomarkers and it has proven to be accurate in the early detection of acute allograft rejection (24). CAD is a complex process with multiple factors that may reduce long-term graft survival. However, urinary proteomics analyses showed that CAD could be predicted as soon as 3 months after transplant by analyzing the urine protein profile and identifying three biomarkers at 8,860 Da (most discriminating), 5,815 Da, and 12,825 Da (sensitivity 83%, specificity 66%, PPV 71%, and NPV 80%). These biomarkers are linked to the degeneration of the tubular cells, which are the first to be affected by chronic allograft dysfunction. However, proteomics analysis can be limited by low levels of proteins and the need to increase the sensitivity and performance of analytical methods (25).

The measurement of targeted proteins in the urine has also been proposed as a way to monitor graft status. Urinary chemokine (C-X-C motif) ligand 9 (CXCL9), an interferon (IFN)-gamma-induced T-cell chemoattractant chemokine released by monocytes/macrophages, endothelial cells, and renal parenchymal cells (16), can non-invasively detect clinical and subclinical acute cellular rejection with a high negative predictive value (17–22). Data from the Clinical Trial in Organ Transplantation 09 (CTOT-09), a study where kidney transplant recipients underwent tacrolimus withdrawal, show that urinary CXCL9 at a positivity threshold of ≥200 pg/ml can detect acute rejection 3–30 days before clinical presentation (26). Data from a case-series indicate that urinary CXCL9 can also be used to monitor the response to therapy for acute rejection (27). Another urinary chemokine, urinary chemokine (C-X-C motif) ligand 10 (CXCL10), has been measured in the urine of kidney transplant recipients and has been shown to outperform CXCL9 in the detection of antibody-mediated rejection (28). An important caveat to consider is that CXCL9 and CXCL10 levels are also elevated during BK virus infection.

Recent studies have suggested that plasma cytokines can predict future allograft rejection in kidney transplantation and could be used as prognostic and monitoring biomarkers. Interleukin (IL)-5 has been recognized as a biomarker linked with short-term stable renal function. However, over time, there is a cytokine signature switch to a pro-inflammatory profile, with a predominance of IL-8, IL-6, IL-1b, tumor necrosis factor-α (TNF-α) and IL-12, especially 10 years after the transplant. IL-12 appears to be the most relevant pro-inflammatory cytokine in long-term kidney transplant recipients (29). However, some studies demonstrated that certain pro-inflammatory cytokines, such as IL-17A and IL-23, can also be used to predict acute allograft rejection (30). At post-transplant day 7, IL-17A was able to detect AR with a sensitivity of 87.5%, specificity of 100%, and AUC of 0.92%. The performance indicators of IL-23 were as follows: AUC, 0.93%; sensitivity, 81.3%; and specificity, 91.1%. It is important to note that the stability of circulating cytokines is variable, and additional research is required to study more thoroughly these biomarkers and validate their application in clinical practice.

### 3.5. Gene expression profiles

The eight-gene assay is a multigene peripheral blood assay used to diagnose AMR. The genes tested include the following: CXCL10; Fc gamma receptor Ia (FCGR1A); Fc gamma receptor Ib (FCGR1B); guanylate-binding protein 1 (GBP1); guanylate-binding protein 4 (GBP4); interleukin 15 (IL-15); killer cell lectin like receptor C1 (KLRC1); and tissue inhibitor of metalloproteinases 1 (TIMP1). In a multiphase multicenter prospective study, Biomarkers of Renal Graft Injuries in kidney allograft recipients (BIOMARGIN), this assay could detect AMR at the time of both graft dysfunction and stable graft function (AUC 79.9%, sensitivity 73.2%, specificity 75.7%, PPV 26.3%, NPV 96.0%), thus capable of detecting subclinical AMR (13). In a follow-up study, the eight-gene assay also demonstrated good diagnostic performance for AMR in both donor-specific antibodies (DSA)-positive and DSA-negative patients (31). The limitations of this assay include an inability to detect TCMR and a decreased accuracy after post-transplant year 1 (13).

The Kidney Solid Organ Response Test (kSORT) is a peripheral blood quantitative PCR assay measuring expression of a 17-gene signature to predict acute rejection. Several included genes are involved in apoptosis regulation, immune phenotype, and cell surface. When tested in a multiphase multinational study with 558 blood samples from 436 renal transplant recipients, the assay was able to predict acute rejection up to 3 months before clinical detection (AUC 0.92, sensitivity 92.31%, specificity 93.48%). Using a novel kSORT algorithm, patients are classified with a numerical score as low risk, high risk, or indeterminate. The test, however, is limited by its inability to discriminate between AMR and TCMR (32). In the Evaluation of Sub-Clinical Acute Rejection PrEdiction (ESCAPE) Study, the kSORT assay and the enzyme-linked immunospot (ELISPOT) assay were evaluated in its ability to predict subclinical AR risk. The kSORT assay, used at the time of a 6-month protocol biopsy, was shown to be an effective rule in test (PPV 93%), but was more effective predicting subclinical AMR compared to TCMR, correctly identifying high risk for 100% and 58% of samples, respectively. The kSORT assay was also tested in combination with an IFN-gamma ELISPOT assay to measure circulating anti-donor reactive T cells (33). The ELISPOT has been shown to predict the risk of acute TCMR at 6-month protocol biopsies (34). The ESCAPE study found the predictive accuracy of detecting subclinical AR, AMR, and TCMR to be significantly improved when using the two assays in combination (33). However, other studies have demonstrated conflicting results on the performance of the kSORT assay. In an independent validation study using the assay in a real-world clinical setting, the test was used on 1,763 blood samples with a concomitant biopsy and showed very little diagnostic value in detecting AR (AUC 0.51) (31). The kSORT assay is clinically available as ImmunoDx, but further investigations are required to determine the accuracy and appropriate clinical uses for the assay.

TruGraf is another peripheral blood gene expression profile capable of establishing an allograft's immune phenotype (20). TruGraf can be used as a non-invasive assessment tool of kidney transplant recipients. It can exclude subclinical acute rejection by analyzing peripheral blood gene expression profiles, with a sensitivity of 71%, specificity of 74%, PPV of 48%, and NPV of 89% (35). As this is a new test, physicians may not be familiar with its use in clinical practice (36). The relatively lower performance of peripheral blood gene expression may reflect the nature of gene signature dilution in the periphery compared to the intragraft microenvironment.

### 3.6. Urinary kidney injury molecule-1

Kidney injury molecule 1 (KIM-1) is a type I transmembrane protein with an immunoglobulin and mucin domain first described by Ichimura et al. in 1998, who noted its upregulation in the proximal tubule cells of post-ischemic rat kidneys (37). Urinary KIM-1 (uKIM-1) is typically undetectable in urine (38). In renal transplant patients, uKIM-1 levels have been observed to be correlated to acute kidney injuries (AKIs) induced by ischemia-reperfusion and associated with cold ischemia time (CIT) (39). There have been discrepancies regarding uKIM-1 as a prognostic biomarker for allograft function. In a single-site, prospective study (*N* = 160), patients with elevated uKIM-1 values on post-transplant day 1 were associated with a higher risk for delayed graft function (DGF) and poorer long-term graft outcomes (40). However, another study found uKIM-1's predictive performance for graft loss (AUC 0.71) to be worse than creatinine and comparable to proteinuria (41). Other studies have found no predictive value for DGF, rejection, or long-term outcomes (39, 42). Additional studies are warranted to determine the potential utility of uKIM-1 in allograft monitoring.

### 3.7. Procalcitonin

Procalcitonin (PCT) is another biomarker of interest in transplant patients. PCT increases in patients with severe bacterial infections (43). This is also observed in kidney transplant recipients, where serum PCT levels dramatically increased during septic conditions, but had little to no change during a localized infection, cytomegalovirus infection, or acute rejection (44). However, PCT has also been studied as a prognostic biomarker for graft failure (AUC 0.84), suggesting that elevated PCT may also reflect chronic, nonmicrobial, low-grade inflammation in the parenchyma of allograft kidneys (45). A later study also found PCT to predict progression to chronic allograft dysfunction (AUC 0.893) in renal transplant recipients (46). It is hypothesized that this elevation in PCT may be caused by its release into circulation by renal parenchymal cells after macrophage infiltration and activation (45).

## 4. Liquid biopsy and biomarkers in kidney diseases

On the other hand, the research efforts on biomarkers are also valuable in native kidney diseases, as they lay the foundations for studies in post-transplant recurrences. Liquid biopsies have been found to be useful in kidney diseases in general. They can be used as a prognostic tool in patients with CKD. The prevalence of CKD indicates that urine proteome analysis (liquid biopsy) can play a significant role in guiding therapeutic goals of care while minimizing discomfort (24). Antibodies against M-type phospholipase A2 (a podocyte membrane glycoprotein) has been found to be associated with primary membranous nephropathy (PMN). Specifically, the anti-phospholipase A2 receptor (anti-PLA2R) antibody was found in 70%–80% of patients with PMN, indicating that this biomarker plays a critical role in the pathogenesis and detection of the disease. Initially, PMN was diagnosed primarily through the detection of proteinuria, and patients were empirically treated through immunosuppressive medications. The detection of elevated levels of anti-PLA2R was found to be associated with a higher risk of nephrotic syndrome, a decreased risk of immunosuppressant-induced remission, and higher rates of end-stage renal disease (ESRD). Low levels of anti-PLA2R antibodies are associated with a higher probability of remission. Furthermore, rituximab (the primary immunosuppressive treatment for PMN) has been found to reduce levels of anti-PLA2R antibodies, which impacts the course of treatment and rate of remission (47). An up-to-date metanalysis involving 19 studies and 1,160 patients investigated the clinical value and accuracy of anti-PLA2R antibodies in relation to diagnostic value and application in clinical practice. The AUC, sensitivity, and specificity of anti-PLA2R was found to be 0.82 (95% CI 0.78–0.85), 68% (95% CI 0.61–0.74), and 97% (95 CI 0.85–1.00), respectively (48). This indicates that anti-PLA2R antibodies have sufficient diagnostic value and should be applicable in diagnostic criteria within the context of patient presentation. However, the detection of PMN using circulating anti-PLA2R antibodies has several limitations. False positives have been described in secondary membranous nephropathy caused by sarcoidosis or hepatitis B virus. anti-PLA2R may also remain positive after the treatment of the autoimmune response. Alternatively, false-negative results can be caused by a high avidity of anti-PLA2R, which lowers the levels of circulating antibodies, and delays their detection until the binding capacity of the podocytes is surpassed (49). Nephrin, a component of the slit diaphragms found in the glomerular foot processes in kidneys, is a crucial component of kidney physiology (50). Loss of the slit diaphragm structure is the primary pathophysiologic mechanism of minimal change disease (MCD). Recent studies have shown that anti-nephrin antibodies have been found in a subset of patients with MCD, indicating there is an autoimmune component in some patients with MCD that can guide treatment options (51). Furthermore, new research is currently being conducted on the clinical association between anti-nephrin and PMN, with some indications that anti-nephrin may be an even better liquid biomarker for PMN than anti-PLA2R antibodies. Anti-PLA2R and anti-nephrin antibodies have proven to be useful biomarkers in the pathophysiology, clinical diagnosis, and course of treatment for nephrotic disease.

Urinary microRNA (mi-106a) has been found to be a potential liquid biomarker for immunoglobulin A (IgA) nephropathy, a post-infectious nephritic syndrome associated with hematuria and proteinuria. IgA nephropathy was found to be associated with six miRNA targets, all of which were significantly elevated throughout the disease progression, indicating that these markers can provide excellent sensitivity for the detection of the disease (52). A research study regarding the urinary miRNA profile for the diagnosis of IgA nephropathy identified 39 miRNA and found that urinary mi-204 had the best diagnostic accuracy. The AUC of the receiving operating characteristic (ROC) for urinary mi-204 in association with the diagnosis of IgA nephropathy was found to be 0.976. The sensitivity and specificity of urinary mi-204 were found to be 100% and 55%, respectively, providing evidence that miR-204 has sufficient accuracy in terms of diagnosis for IgA nephropathy (53).

Liquid biomarkers have also been found to play a role in the detection of the early stages of hypertensive and diabetic glomerulonephropathy, indicating its significance as a non-invasive method of diagnosis in association with comprehensive urine and blood analysis (54). The analysis of urinary peptide content (urinary peptidomic biomarkers) has also been associated with the early detection of nephrotic syndromes and has been found to improve targeted pharmacologic therapy.

Finally, exosomes are also a promising tool in predicting the onset and progression of certain kidney diseases, such as AKI, CKD—especially diabetic nephropathy, lupus nephritis, polycystic kidney disease (PKD), renal cell carcinoma (RCC), and MCD. However, techniques still need to be optimized and standardized. Research is still needed to improve the accuracy, reliability, and reproducibility of the results (19).

## 5. The need for biopsies and challenges

Surveillance biopsy may be performed to detect subclinical acute rejections, especially in patients undergoing major changes in immunosuppression regimens. However, they have several disadvantages: they are inconvenient, expensive, subjective in interpretation, and carry potential complications (bleeding, hematoma, etc.) (5). Moreover, there is no optimal timing or frequency for surveillance biopsies. Biomarkers and liquid biopsies can overcome these limitations as they are non-invasive and reduce the rate of unnecessary biopsies. At the present time, a number of the previously discussed biomarkers can help in reducing biopsies, such as ddcfDNA, if used appropriately. However, more research is needed to conclusively identify patients who should be biopsied, and to implement new practices in clinical settings (6).

Various challenges need to be overcome for the widespread use of liquid biopsy biomarkers in kidney diseases. Their performance is affected by many factors (age, ethnicity, pre-existing conditions, etc.); it is therefore important to ensure adequate study populations and proper controls (55). Furthermore, kidney diseases are extremely heterogeneous, which can be a major challenge for biomarkers to be used in a clinical setting. Tissue biopsies are often required to identify important pathological features in kidney diseases, and specifically in kidney transplant recipients.

On the other hand, methodological and technical limitations should be taken into account: The biomarker levels and stability can vary based on the sample's type and disease stage. They are often in low amounts and a considerable sample volume is critical to detect them (56). The assays also need a high level of sensitivity to identify circulating molecules and reduce the rate of false-negative results. Highly sensitive methods are being developed as a solution but are at risk of false positives (e.g., ultrasensitive ctDNA assays can give a false-positive result from normal white blood cell contamination). Large, multicenter clinical studies are therefore still needed to overcome these challenges. Finally, the pre-analytical phase of the liquid biopsy—specimen collection, processing, stabilization, transport, enrichment, and storage—is crucial in determining its validity. The major challenge here is to preserve the biomarkers that will be studied and maintain the integrity of other components to prevent contamination. However, standardized pre-analytical criteria have not been universally defined, apart from some norms, such as preventing hemolysis and blood clotting. All these assays need to be uniformed in terms of sample extraction procedure, processing, and isolation, to obtain comparable data and draw meaningful conclusions (57).

The creation of consortia speeds up the research on liquid biopsies and contributes to the standardization of collection protocols and strategies. It also comes as a solution for the limited number of samples each center has, and the small genetic variability of the cohorts. Joint efforts help transfer the findings from bench to bedside, to allow a much-needed use of biomarkers in clinical practice, especially in kidney diseases. The Liquid Biopsy Consortium for Kidney Diseases (LICUID) created in 2022 comes as an answer to help advance precision medicine for patients with kidney diseases and kidney transplant recipients. The priority of this consortium is to develop liquid biopsy techniques by sharing biological specimens between experts from around the world and ensuring that standardized biobanking procedures are respected. The findings validity is guaranteed by providing an important number of high-quality samples and allowing data validation in multiple collaborating centers. The LICUID website (www.licuidconsortium.com) can also serve as a valuable reference resource, outlining standardized protocols for liquid biopsies.

## 6. Conclusion

Several diagnostic, prognostic, and predictive biomarkers are being developed, due to the lack of necessary tools to identify native and transplant kidney disease, with the need to define universal pre-analytical standards. Table 1 provides a comprehensive summary of each biomarker's characteristics. While there is a myriad of challenges with biomarker research, establishing consortia such as LICUID is a promising solution, allowing for a widespread collaboration across the globe. In doing so, the sample size that would take one site alone years to gather, can be gathered and analyzed at an expedited rate. The LICUID consortium aims to achieve this task through vast collaboration on liquid biopsy techniques in kidney disease and transplantation research. To learn more about LICUID, visit www.licuidconsortium.com.

**Table 1:** Characteristics of biomarkers in kidney transplants.

Biomarker	Advantages	Disadvantages	Sample	AUC	Sensitivity	Specificity	PPV	NPV	Notes
ddcfDNA (16)	Superior to creatinine in allograft rejection assessment	Injury signature, not specific for rejection. False negative in TCMR Ia	Plasma	0.74	59%	85%	61%	84%	
Exosomal mRNA (20, 21)	Stable. Identify all cause rejection and can distinguish between TCMR/AMR	Expensive, not widely available. Large validation studies still needed	Urine	0.93	NA	NA	86.2%	93.3%	
Exosomal miRNA (22)	miR-21, miR-210 and miR-4639 can detect CAD	Expensive, not widely available. Large validation studies still needed	Plasma	0.89	88.46%	73.08%	NA	NA	
mRNA (granzyme B, perforin, FasL, FOXP3) (14)	Can detect acute allograft injury from plasma or urine	Unstable. Large validation studies needed	Plasma/urine	NA/0.95	100%/94%	95%/95%	100%/94%	95%/95%	
miRNA-210 (14)	Stable (compared to mRNA). Can predict acute allograft rejection	No standard protocol for isolation and processing	Urine	0.70	52%	74%	NA	NA	
Proteomics (8860 Da, 5815 Da, 12825 Da) (24, 25)	Can predict CAD as soon as 3 months after transplant	Low levels, analytical methods need improvement	Urine	NA	83%	66%%	71%	80%	
IL-17A/IL-23 (29)	Can predict Acute allograft injury	Variable stability. Additional research required	Plasma	0.92/0.93	87.5%/81.3%	100%/91.1%	NA	NA	
Eight gene assay (13)	Detection of subclinical AMR	Inability to detect TCMR and a decreased accuracy after the first year post-transplant	Plasma	0.79	73.2%	75.7%	26.3%	96%	
kSORT (32)	Predict subclinical acute rejection, up to 3 months prior to clinical detection	Inability to discriminate between AMR and TCMR	Plasma	0.92	92.31%	93.48%	NA	NA	
TruGraf (35)	Can exclude subclinical acute rejection	New test, unfamiliar with physicians for clinical use	Plasma	NA	71%	74%	48%	89%	
KIM-1 (41)	In transplant patients, it is correlated to AKIs induced by ischemia-reperfusion and associated with cold ischemia time	Variable study outcomes. More research is still needed	Urine	0.71	NA	NA	NA	NA	
PCT (45)	Predict chronic allograft dysfunction	Not specific	Plasma	0.84	NA	NA	NA	NA	
Anti-PLA2R (48, 49)	Detection of PMN. Correlated with a higher risk of nephrotic syndrome, higher rates of ESRD and decreased risk of immunosuppressant-induced remission	False positives in some secondary causes of membranous nephropathy. False negatives due to a high avidity of antibodies	Plasma	0.82	68%	97%	NA	NA	
mi-204 (53)	Detection of IgA nephropathy	No standard protocol for isolation and processing	Urine	0.976	100%	55%	NA	NA	

AUC, area under the curve; PPV, positive predictive value; NPV, negative predictive value; TCMR, T-cell mediated rejection; AMR, antibody-mediated rejection; CAD, chronic allograft dysfunction; ddcfDNA, donor-derived cell-free DNA; mRNA, messenger RNA; miRNA, microRNA; kSORT, Kidney Solid Organ Response Test; KIM-1, Kidney Injury Molecule 1; PCT, procalcitonin; AKI, acute kidney injury; PMN, primary membranous nephropathy; ESRD, End Stage Renal Disease.
